# Effect of Motivation by “Instagram” on Adherence to Physical Activity among Female College Students

**DOI:** 10.1155/2016/1546013

**Published:** 2016-02-29

**Authors:** Einas Al-Eisa, Asma Al-Rushud, Ahmad Alghadir, Shahnawaz Anwer, Bashayer Al-Harbi, Noha Al-Sughaier, Noha Al-Yoseef, Reem Al-Otaibi, Hanadi Ali Al-Muhaysin

**Affiliations:** ^1^Rehabilitation Research Chair, College of Applied Medical Sciences, King Saud University, Riyadh 11433, Saudi Arabia; ^2^Department of Rehabilitation Sciences, College of Applied Medical Sciences, King Saud University, Riyadh 11433, Saudi Arabia; ^3^Dr. D. Y. Patil College of Physiotherapy, Dr. D. Y. Patil Vidyapeeth, Pune 411026, India

## Abstract

*Objective.* To investigate the efficacy of using “Instagram application” with a “home-exercise program” as a motivational stimulus in improving physical activity (PA) adherence levels among female college students.* Methods.* Fifty-eight female undergraduate students with the mean age 20.3 ± 0.96 years participated. Participants were divided into two groups: intervention and the control group; both the groups received an exercise program and the intervention group was additionally motivated by “Instagram.” Adherence to PA was measured by using an adherence sheet. The Exercise Motivation Inventory (EMI-2) was used to assess the motivational factors.* Results.* The most frequent motivational factors were extrinsic as assessed using the EMI-2. “Positive health” was the most frequent factor mentioned of the two types with 47% of the sample. The intervention group adhered with 17% more to the activity program compared to the control group. Moreover, 72% of the participants in the intervention and control groups found the activity program flexible enough to be performed at home; they agreed about its effectiveness on adherence (53%).* Conclusions.* The use of Instagram with the home exercise program as a motivational modality could be attractive and effective to reinforce adherence and maintain an appropriate PA level.

## 1. Introduction

Adherence to physical activity (PA) leads to improvements in physical function and quality of life [1]. The adherence is one of the most important factors in order to get the desired benefits of PA. The role of the PA has been well-discussed in several studies and found to aid prevention of many health disorders, such as cardiovascular diseases and obesity [2, 3]. Regular exercise, in particular, helps protect against debilitating and costly chronic conditions as well and to achieve the optimum level of quality of life [4].

Adherence to PA must be improved in order to improve health prospects. The adherence to the PA has been found to be extremely low in the Saudi population, particularly among young females [5]. This poor adherence could be attributable to lack of knowledge about the benefits of exercise or even many related social and personal factors as well as cultural factors [6]. The dangers of physical inactivity have been outlined in many studies as it is one of the most important public health problems of the 21st century [7]. For example, physically inactive middle-aged women (performed less than 1 hour of exercise per week) experienced 52% increased all-cause mortality, a doubling of cardiovascular related mortality and 29% increased cancer-related mortality compared with the physically active women [8].

Motivation for PA could be defined as the intrinsic and extrinsic factors that stimulate desire and energy in people to be continually interested and committed to the initiation and the maintenance of a PA program or any type of exercise [9]. Intrinsically motivated behaviors are those performed for satisfaction one gains from engaging in the activity itself. By contrast, extrinsically motivated behaviors are those performed in order to get rewards or results that are separate from the behavior itself [10]. There are many different ways that have been used to increase the adherence level to the PA and the home exercise programs are an effective motivational way [11]. Previous studies reported positive effect of using electronic media and mobile phone applications to enhance motivation in improving adherence among participants [12–14]. Fukuoka et al. [12] investigated the use of mobile phone based intervention to improve physical activity in sedentary women. They concluded that the mobile phone based intervention seems to motivate inactive women to improve their physical activity. Another study reported significant effects of Internet and mobile phone based motivation to improve the level of physical activity in healthy adults [13]. In addition, Turner-McGrievy et al. reported some potential benefits of mobile phone monitoring methods during weight reduction intervention in overweight adults [14].

The present study aimed primarily to investigate the efficacy of using social media “Instagram application” with a “home exercise program” as a motivational stimulus in improving PA adherence levels among Saudi female college students and secondarily to measure the motivational factors that influences their engagement or continued participation in an exercise program.

## 2. Methods

Fifty-eight undergraduate female students from the College of Applied Medical Sciences (CAMS), King Saud University, Riyadh, participated in this quasi-experimental study. Self-selection sampling method was used. The selected sample was mostly newcomers to exercise and the time of study was in the second semester of the academic year and the designed program started before the middle of the semester at the period of first midterm exams. They were assigned into two groups in a nonrandomized way. The inclusion criteria were as follows: the participants should have online social network (Instagram) accounts, female students in the age range from 18 to 25 years. The participants were excluded if they are pregnant and had any medical problems. The purpose and procedure of the study and the activity program were explained and demonstrated to each participant. Each participant who agreed to participate signed written informed consent approved by the institutional ethics committee.

Anthropometric parameters including body weight, height, and body mass index (BMI) were measured. The participants wore simple clothing and had no footwear. Height was measured using a fixed stadiometer (resolution of 0.5 cm). The weight was measured with Beurer glass diagnostic scale (resolution of 0.1 kg). The BMI was estimated using the formula: weight (kg)/height (m)^2^. Each participant asked to respond to the Exercise Motivation Inventory-2 (EMI-2) questionnaire [15, 16]. The EMI-2 is made up of 51 items that constitute 14 subscales, which provide extensive measure of motivation to engage in physical activity.  Table 1 details the lists of subscales, number of items, and sample questions. Each item was answered on a 5-point scale ranging from 0 (not at all true for me) to 5 (very true for me) [15]. In addition, an exercise video which is freely available on the YouTube (https://www.youtube.com/watch?v=fcN37TxBE_s) was played which consisted of 37 minutes of cardio workout. This workout was lower to moderate intensity and is made for the beginners. The participants were divided into two groups the intervention group (Instagram group) and the control group. The intervention group had an online social network (Instagram) accounts; however, control group had no such account. An email was sent to all of the participants about the date for returning the adherence sheet and only the intervention group received the study account in Instagram and was required to follow it. A variety of techniques were used in Instagram to improve motivation for adherence to PA. First, a small number of pictures were posted to educate the participants about the benefits of PA such as benefits of exercise on bone density, back pain, stress, and mood. Secondly, Instagram was used as an alarm, to remind them to do the exercise sessions at each week. They asked to post their own pictures of adherence sheet when they complete the session in order to motivate other participants to do the exercise. In addition, they received the exercise program video link (can be accessed on YouTube https://www.youtube.com/watch?v=fcN37TxBE_s); this was an exercise video from Fitness Blender Cardio Workout at Home, which was a 37-minute, Fat Burning Cardio Workout, 3 in difficulty. It focused mainly on the lower body and was also generally for all the body. It consists of a warmup, workout, and a cool down at the end of the video. This activity is designed to be performed at home without any need for any exercise equipment or registration in a gym and this type of activity was moderate intensity physical activity, 150 minutes per week according to the 2008 Physical Activity Guidelines for Americans [17]. After the period of intervention, which was 4 weeks, a feedback questionnaire was collected to investigate the flexibility, effectiveness, and motivation level for given program activity.

The Statistical Package for the Social Sciences (SPSS) program “version 22” and Microsoft Office Excel 2007 were used in the statistical analysis. The unpaired *t*-test was chosen to compare adherence between the two independent groups (intervention and control groups). Descriptive statistics for the motivational factors and the flexibility and effectiveness of the program were employed to analyze the data. A level of significance was taken *P* < 0.05.

## 3. Results

Fifty-eight students completed the EMI-2 survey, and 47 of 58 were included in the intervention and control groups to test the efficacy of intervention. Eleven participants did not submit their adherence sheet (attrition rate 19%).  Table 2 shows the general characteristics of the study sample, the total number of study participants amounted to 58 unmarried females. According to the World Health Organization BMI classification, 12% of the participants were underweight and only 3% were categorized as obese.  Table 3 shows the important extrinsic and intrinsic motivational factors. Forty-seven percent of the students were motivated to gain positive health and 2% by social recognition and health pressure.  Table 4 reflects the difference between the two groups with regard to adherence to the exercise program, according to the number of sessions that had been done in 4 weeks. Only 4% of the control group was adherent to overall 4 weeks and there was 17% adherent in the intervention group. There is a significant difference between the two groups (*P* = 0.04).  Table 5 presents participant's feedback toward the given activity program, 34% found it flexible, 25% found it effective, and 30% felt motivated by this program.  Table 6 shows the result of the second section of the questionnaire which reflects the nonadherence reasons. Twenty students indicate academic stress as a reason while only one student felt not motivated by the program.

## 4. Discussion

The primary aim of this study was to investigate the efficacy of using social media “Instagram application” with a “home exercise program” as a motivational stimulus in improving PA adherence level. It has been hypothesized that motivation by using “social media” in combination with an “Organized Activity Program” would increase adherence to regular PA. Previous studies suggested that the use of electronic media to improve motivation had an effect in improving adherence among participants [12–14].

In the present study, participants from the intervention group performed more than eight sessions compared to control group who performed 2-3 sessions only; this might be referred to the motivation caused by Instagram in the intervention group. These findings were consistent with those observed in the previous study [13], who reported increased accelerometer-measured PA in the Internet and mobile phone based PA group. Similarly, the results of a recent meta-analysis of randomized controlled trials [18] reported that the social media may provide certain advantages for public health interventions. It can be suggested that using social media to motivate people to perform an exercise program could have a positive influence on individual's adherence to regular PA.

From the above discussion it follows that adherence is a subjective characteristic that all people have in different levels and it is affected by many social and personal factors that must be considered in any situation [19]. Therefore, measuring the most important motivational factors that influence participants' behavior to engage or continue with an exercise program was the second aim of the present study. The results of present study showed that the extrinsic motivational factors, which were mentioned with high frequency, are “positive health,” “ill-health avoidance,” and “weight management and nimbleness,” while intrinsic factors were mentioned less frequently and this could be related to the selected sample which were female students who have concerns for health and its related factors as they are students of medical specialties. Similarly, disease prevention, physical fitness, body weight management, and stress management were the commonest factors reported in the previous study to improve motivation for exercise in university students [20]. In addition, females are very often concerned about body weight and being agile and this consisted in another study conducted among active college students, which observed a similar pattern of scores on nimbleness, with women considering this the most important motive for exercise [21]. Moreover, it can be said that the extrinsic factors were more often observed as motives for exercise while the intrinsic factors were more related to the sports participation [10].

Participants were asked to perform exercise sessions as they feel and want to get benefits of PA, so they could continue in any type of regular PA. Adherence to certain exercise programs is predicted by using of motivation in the program, and it is an effective way to encourage a person to participate in the activity [22]. In the present study, motivation by exercising at home was used. From the reported findings, it can be said that the given physical activity program was sufficiently flexible to be performed at home without the need to register with a fitness gym and use any exercise equipment, or any specific recruitments of exercise. Therefore, exercising at home could improve adherence to regular physical activity, especially if the program is combined with a self-reported adherence sheet which can give the trainee more acceptance with and confidence in the program. In addition, there may be an encouragement for adherence to exercise due to the positive feelings related to the accomplishment achieved during the sessions. These results are comparable with the previous study who found that self-monitoring of home exercise is effective in improving exercise adherence and self-efficacy [11].

Moreover, investigating the flexibility and effectiveness of the designed home exercise program to improve adherence to PA was an objective of the current study. The participants' opinions about the motivation for PA with the designed program were mostly positive in comparison to their adherence to the program which was slightly negative. This may indicate that the program was effective in a motivational way to encourage participation in the activity program but for adherence it was insufficient. Some reasons for nonadherence to the exercise program were given by the participants and the three most important were “academic stress, not enough time,” and “the program is hard to perform” and “not motivated by the program” was also mentioned. In the previous study, the lack of time was one of the commonly reported reasons for nonadherence [23]. Another study reported poor adherence for high-intensity PA compared to low-intensity exercise [24]. Abdul Salam and Siddiqui have investigated the social-demographic determinants of compliance among Type-2 diabetic patients and they concluded that the Saudi patients were significantly compliant with medication while non-Saudis were compliant with exercise [25]. In addition, adherence to treatment and self-care activities such as diet and exercise was also identified as adherence predictors among diabetic patients [26]. The often sedentary lifestyle of the Saudi population and the lack of a culture of exercise and deep knowledge about PA benefits were also thought to be strongly related to the previous reasons for nonadherence to PA. Previous studies reported a high rate of physical inactivity in Saudi populations [5, 27]. Finally, it is well known that health beliefs and cultural influences are thought to be factors related to adherence levels among any population [28]. In addition, there is a causal relationship between health beliefs and the level of physical exercise [29]. Additionally, cultural influences may lead to physical inactivity among Saudi women [6].

The present study had some potential limitations. The sample size was small due to the short duration of study time. If the study had included multiple colleges on the campus, diverse findings might have emerged. The study required to receive adherence sheets about the activity program from all included participants. This being said, there were 11 participants of 58 who did not return the sheets and there were 2 participants excluded from the study which resulted in a 23% attrition rate from the recruited sample (*n* = 60) and some of the study findings may have been affected by this. In addition, sample was selected based on self-selection technique instead of probability sampling, which could affect the results. For future research, it is recommended that further investigation in this field needs to be undertaken in different colleges on the campus as well as at various universities, and with a sample which includes people with different levels of education, and increased duration of intervention to test adherence to PA program more accurately. Home based exercise is a good motivational way to participate in physical exercise, but it was found insufficient for exercise adherence. Thus more effective modifications to this type of motivation may well improve adherence. Health providers and trainers at the health centers should activate the use of social network sites to provide guidance, instruction, and motivation to the people to maintain regular PA in order to gain a healthy lifestyle.

## 5. Conclusions

The adherence to PA among female college students was poor. The use of Instagram with the home exercise program as a motivational modality could be attractive and effective to reinforce adherence and maintain appropriate PA levels among these populations.

## Figures and Tables

**Table 1:** The exercise motivation inventory-2 questionnaire.

Subscales	Number of items	Sample questions
Stress management	4	To release tension
Revitalization	3	To recharge my batteries
Enjoyment	4	To enjoy the social aspects of exercising
Challenge	4	To give me personal challenges to face
Social Recognition	4	To gain recognition for my accomplishments
Affiliation	4	To spend time with friends
Competition	4	Because I like trying to win in physical activities
Health pressures	3	To help prevent an illness that runs in my family
Ill-health avoidance	3	To avoid ill-health
Positive health	3	To feel more healthy
Weight management	4	To help control my weight
Appearance	4	To improve my appearance
Strength and endurance	4	To get stronger
Nimbleness	3	To stay/become flexible

**Table 2:** Demographic data of the participants.

	Mean ± SD	Percentage (100%)
Age (years)		
19	20.30 ± 0.96	20%
20		47%
21		18%
22		15%
Weight (kg)	58.80 ± 13.04	
Height (cm)	158.1 ± 5.88	
BMI (kg/m^2^)		
Underweight <18.5	23.6 ± 4.72	12%
Normal 18.5–24.99		60%
Overweight ≥25		18%
Obese ≥30		3%

**Table 3:** Frequency and percentage of the extrinsic and intrinsic motivational factors.

Motivational factors (subscale)	Number of items	Sample items	Frequency	Percentages
Stress management	4	Because it helps reduce tension	7	12%
Revitalization	3	Because it makes me feel good	0	0%
Enjoyment	4	Because I enjoy the feeling of exerting myself	7	12%
Challenge	4	To give me goals to work toward	7	12%
Social recognition	4	To show my worth to others	1	2%
Affiliation	4	To spend time with friends	0	0%
Competition	4	Because I like trying to win in PA	2	3%
Health pressures	3	Because my doctor advised me to exercise	1	2%
Ill-health avoidance	3	To prevent health problems	21	36%
Positive health	3	To have a healthy body	27	47%
Weight management	4	To stay slim	18	31%
Appearance	4	To look more attractive	11	19%
Strength & endurance	4	To increase my endurance	13	22%
Nimbleness	3	To stay/become more agile	16	28%
Total	51		58	100%

**Table 4:** Difference between the participants in adherence to the activity program.

Classification	Number of sessions	Control group	Intervention group
Nonadherent	0–4	88%	61%
	5–8	8%	22%
Adherent	≥8	4%	17%
Mean ± SD		2.09 ± 2.29	3.83 ± 4.05
*P* value		0.04^*∗*^	

^*∗*^Significant at *P* < 0.05.

**Table 5:** Measuring the flexibility, effectiveness, and motivation of the activity program.

Feedback of the program activity	Program was flexible	Program was effective	Program was motivating
Yes	34 (72%)	25 (53%)	30 (64%)
No	7 (15%)	8 (17%)	8 (17%)
Indifferent	6 (13%)	14 (30%)	9 (19%)

**Table 6:** Reasons of nonadherence to the activity program.

Reasons	Frequency (%)
Academic stress	20 (42.5)
Not enough time	12 (25.5)
Program is hard to perform	4 (8.5)
Not motivated by program	1 (2.1)
Others	10 (21.3)

## Abbreviations

EMI-2: The Exercise Motivation Inventory-2 questionnaire
PA: Physical activity
BMI: Body mass index.
